# Are the CDC’s *Inside Knowledge* Campaign Educational Materials Being Used to Increase HIV-Positive Women's Knowledge about Human Papillomavirus and Cervical Cancer? A Narrative Review

**DOI:** 10.1089/jwh.2018.7453

**Published:** 2019-02-23

**Authors:** Lisa T. Wigfall, Rossmary Marquez-Lameda

**Affiliations:** ^1^ Division of Health Education, Department of Health and Kinesiology, College of Education and Human Development, Texas A&M University, College Station, Texas.; ^2^ *Éxito!* Latino Cancer Research Leadership Training Program, Institute for Health, Promotion Research, UT Health San Antonio, San Antonio, Texas.

The main purpose of the Centers for Disease Control and Prevention (CDC) *Inside Knowledge* campaign launched in 2008 is to increase women and health care providers' knowledge about five gynecological cancers (cervical, ovarian, uterine, vaginal, and vulvar cancers).^1^ Human papillomavirus (HPV), a common sexually transmitted infection, causes three of these gynecological cancers (cervical, vaginal, and vulvar).^2–4^

Although HPV infection is transient in most women, HIV-positive women are three times more likely to develop cervical cancer (an AIDS-defining illness) because their weakened immune system is less able to clear HPV infection.^5–7^ Some researchers have shown that cervical cancer screening (*i.e.*, Pap tests) is underutilized among HIV-positive women who are at greatest risk for developing cervical cancer (*i.e.*, older and low CD4 cell counts).^8^ Despite these disparities and increased risk, some HIV-positive women have low knowledge about HPV and cervical cancer,^9,10^ which includes being unaware of the fact that HIV infection increases their risk of developing cervical cancer.^7^ Even less is known about other HPV-related gynecological cancers (*i.e.*, vaginal and vulvar cancers).

We examined whether or not the *Inside Knowledge* campaign educational materials are being used to increase HIV-positive women's knowledge about cervical cancer. We combined (((“inside knowledge”) and gynecologic*) and cancer) to search CINAHL Complete, PubMed/MEDLINE Complete, PsycINFO (*n* = 2) and Web of Science research databases for studies about the CDC's *Inside Knowledge* campaign. The asterisk (*) is used as a wildcard to include “gynecologic” and “gynecological”. Publications (*n* = 20) listed on the *Inside Knowledge* campaign research webpage were hand searched.^1^ Twenty-four de-duplicated titles/abstracts were reviewed by the authors.

**FIG. 1.**
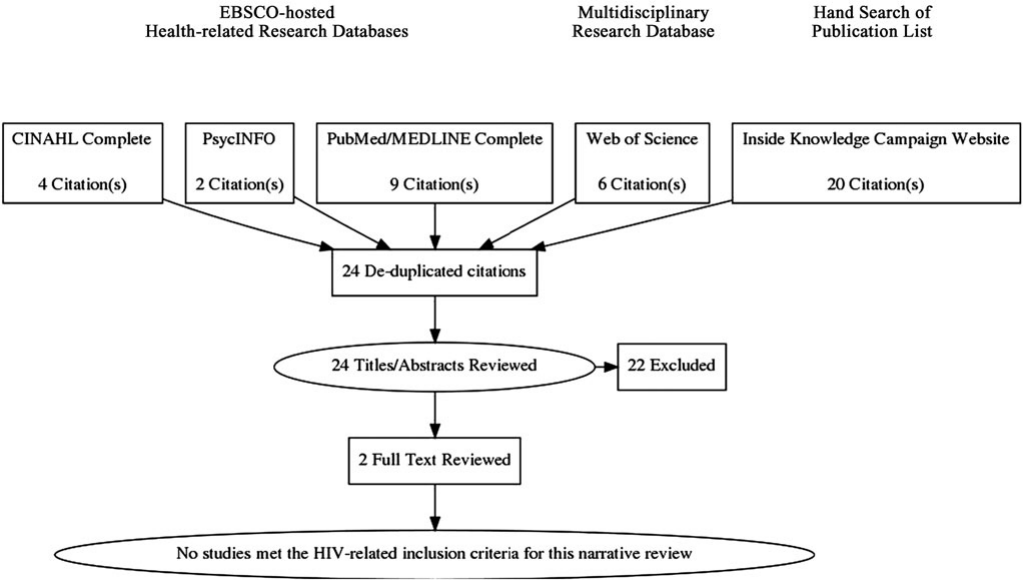
PRISMA flow diagram. PRISMA, Preferred Reporting Items for Systematic Reviews and Meta-Analyses.

Included studies had to describe an intervention that used the *Inside Knowledge* campaign educational materials to (1) increase HIV-positive women's knowledge about cervical cancer or (2) raise awareness about HIV infection as a cervical cancer risk factor. Three studies were educational interventions, including one about ovarian cancer. The full text of the remaining two studies were electronically searched and “HIV” was not found. No studies met our inclusion criteria (Fig. 1).

Almost 10 years after the CDC's launch of the *Inside Knowledge: Get the Facts About Gynecologic Cancer* campaign, there are only a few published studies that used these evidence-based educational materials to increase women and health care providers' knowledge about gynecological cancers (*i.e.*, cervical, ovarian, uterine, vaginal, and vulvar). Although no studies were included in our narrative review of the *Inside Knowledge* campaign research literature, it is our opinion that the absence of HIV infection as a cervical cancer risk factor^5,7^ and HIV-positive women's need for cervical cancer prevention education^9,10^ in both of these studies was noteworthy. To this end, it is our view that HIV-positive women should be included in evidence-based cervical cancer prevention educational interventions (ideally) and awareness raised about the link between HIV infection and cervical cancer risk (minimally).^5,7^

HPV vaccination is understudied among HIV-positive women. Although some researchers have studied cervical cancer screening-related outcomes among HIV-positive women in the United States,^10^ most of these studies have been conducted in low- and middle-income countries despite the fact that HIV-positive women in the United States continue to be disproportionately affected by cervical cancer.^7^ Our study underscores the need for culturally appropriate evidence-based cervical cancer prevention educational programs that target HIV-positive women with information that is tailored to their specific health needs. This unmet need for evidence-based cervical cancer prevention educational programs becomes increasingly important for at-risk populations such as HIV-positive women, who, similar to their HIV-negative female counterparts, not only need to be educated about cervical cancer risk factors, symptoms, and effective prevention tools (*i.e.*, HPV vaccine, Pap test, and HPV DNA test), but also especially need to be made aware of the fact that HIV infection increases their risk of developing cervical cancer.^7^

The *Inside Knowledge* campaign educational materials are among a few resources that include HIV infection as a cervical, vaginal, and vulvar cancer risk factor.^5^ For that matter, the *Inside Knowledge* campaign educational materials are also among a few resources about other HPV-related gynecological cancers (*i.e.*, vaginal and vulvar cancers).^5^ Raising awareness is an intervention strategy that was used in 19 of 20 evidence-based HPV vaccination and/or cervical cancer screening programs that were listed on the National Cancer Institute's Research-Tested Intervention Programs website.^11^ More research is needed to better understand how to increase HIV-positive women and HIV care providers' knowledge about cervical cancer and other HPV-related gynecological cancers (*i.e.*, vaginal and vulvar cancers).^9,10^ One innovative approach to increasing HIV-positive women's knowledge about HPV-related gynecological cancers (*i.e.*, cervical, vaginal, and vulvar cancers) is to explore engaging community-based HIV/AIDS service organizations in the CDC's efforts to disseminate the *Inside Knowledge* campaign educational materials to at-risk disparate target populations such as HIV-positive women.^4^
